# Using the collaborative intervention planning framework to adapt a health-care manager intervention to a new population and provider group to improve the health of people with serious mental illness

**DOI:** 10.1186/s13012-014-0178-9

**Published:** 2014-11-30

**Authors:** Leopoldo J Cabassa, Arminda P Gomes, Quisqueya Meyreles, Lucia Capitelli, Richard Younge, Dianna Dragatsi, Juana Alvarez, Yamira Manrique, Roberto Lewis-Fernández

**Affiliations:** School of Social Work, Columbia University, 1255 Amsterdam Avenue, New York, NY 10027 USA; New York State Psychiatric Institute, Room 3206, Unit 69, 1051 Riverside Drive, New York, NY 10036 USA; Columbia University Medical Center, 100 Haven Suite 27C, New York, NY 10032 USA

**Keywords:** Intervention adaptation, Community engagement, Implementation science, Serious mental illness, Health-care managers, Health disparities

## Abstract

**Background:**

Health-care manager interventions improve the physical health of people with serious mental illness (SMI) and could be widely implemented in public mental health clinics. Local adaptations and customization may be needed to increase the reach of these interventions in the public mental health system and across different racial and ethnic communities. In this study, we describe how we used the collaborative intervention planning framework to customize an existing health-care manager intervention to a new patient population (Hispanics with SMI) and provider group (social workers) to increase its fit with our local community.

**Methods:**

The study was conducted in partnership with a public mental health clinic that serves predominantly Hispanic clients. A community advisory board (CAB) composed of researchers and potential implementers (e.g., social workers, primary care physicians) used the collaborative intervention planning framework, an approach that combines community-based participatory research principles and intervention mapping (IM) procedures, to inform intervention adaptations.

**Results:**

The adaptation process included four steps: fostering collaborations between CAB members; understanding the needs of the local population through a mixed-methods needs assessment, literature reviews, and group discussions; reviewing intervention objectives to identify targets for adaptation; and developing the adapted intervention. The application of this approach enabled the CAB to identify a series of cultural and provider level-adaptations without compromising the core elements of the original health-care manager intervention.

**Conclusions:**

Reducing health disparities in people with SMI requires community engagement, particularly when preparing existing interventions to be used with new communities, provider groups, and practice settings. Our study illustrates one approach that can be used to involve community stakeholders in the intervention adaptation process from the very beginning to enhance the transportability of a health-care manager intervention in order to improve the health of people with SMI.

**Electronic supplementary material:**

The online version of this article (doi:10.1186/s13012-014-0178-9) contains supplementary material, which is available to authorized users.

## Background

Implementing health-care interventions in public mental health clinics is a pressing need since people with serious mental illness (SMI) face persistent health disparities. Integrating medical and behavioral health services for people with SMI is considered a key strategy to address these health inequities [1,2]. Few studies currently exist describing the systematic steps that can be used to adapt health-care interventions for routine practice in public mental health clinics. Clinic directors, mental health providers, peer advocates, and researchers involved in the integration of medical and behavioral health services for people with SMI could benefit from systematic intervention planning approaches that help them customize health-care interventions to their local context. In this article, we describe how we used the collaborative intervention planning framework, an approach that combines community-based participatory research (CBPR) principles and intervention mapping (IM) procedures [3], to inform the adaptation of a health-care manager intervention to a new population (Hispanics with SMI) and provider group (social workers) to enhance its use in public mental health clinics.

People with SMI die at a younger age than the general population largely due to elevated rates of cardiovascular disease (CVD) and other chronic medical conditions [4,5]. Racial/ethnic minority status may contribute additional health risks for people with SMI. Hispanics with SMI have elevated rates of obesity, metabolic syndrome, and type 2 diabetes compared to non-Hispanic Whites with SMI [6-8]. Poor access to primary care, fragmented care between mental health-care and primary care providers, and poor quality health care contribute to these disparities [1]. Health-care manager interventions designed to improve care coordination, patient education, and activation can help alleviate these inequities in primary care [9]. For example, the Primary Care, Access, Referral and Evaluation (PCARE) program uses registered nurses (RNs) as health-care managers in community mental health clinics and has been shown to significantly increase the rate of preventive primary care, improve the quality of care for cardiometabolic conditions (e.g., hypertension), and improve mental health-related quality of life among adults with SMI, primarily African Americans, compared to usual care [10].

Given these results, health-care manager interventions like PCARE could be more widely implemented in public mental health clinics to address the health and health-care disparities faced by people with SMI. An important step for expanding the use of these interventions is to customize them to other populations and provider groups in order to enhance their reach within the SMI population. This step requires some level of intervention adaptation to the local context since the introduction of a new intervention rarely fits perfectly within the organization and community in which it is being implemented [11]. The goal of our study is to describe how we applied the collaborative intervention planning framework to inform the adaptation of PCARE to address the needs of Hispanic clients with SMI and facilitate social workers’ delivery of this intervention without compromising its core ingredients.

## Methods

### Setting

This study was conducted at a public mental health clinic in New York City. The catchment area for this clinic is approximately 71% Hispanic, mostly of Dominican descent, and about half of the residents are foreign-born [12]. Approximately, 31% of residents live in poverty and 20% lack health insurance [12]. One in five adults in these neighborhoods is obese, and chronic medical conditions (e.g., diabetes) are prevalent [12]. This clinic serves people with SMI that reside in this community. In 2013, the clinic served 1,100 clients. More than half of the clients are overweight/obese, and the most common medical conditions include diabetes, high cholesterol, and hypertension [6]. All participants gave written informed consent to participate in this study, and all research procedures were approved by the institutional review boards from the New York State Psychiatric Institute and Columbia University.

A key priority identified by the clinic’s leadership at the beginning of this project was to improve the clients’ links to primary care and ensure proper coordination of primary care services. A health-care manager intervention was chosen for several reasons. First, limited resources prevented the clinic from employing and embedding a primary care provider to address the clients’ health-care needs. Second, health-care manager interventions are recommended by the Institute of Medicine [1] as a critical approach to improve the health of people with complex physical and mental health conditions. Lastly, these interventions are suitable for public mental health clinics since different providers (e.g., nurses, social workers) can take on the health-care manager role. Since social workers outnumbered nurses at this clinic, they were considered a natural fit for the health-care manager role since they worked closely with minority patients and bring expertise in counseling and system navigation that match the skills required for effective care management.

### Health-care manager intervention

The intervention being adapted in this study is based on PCARE, a 12-month intervention in which health-care managers (e.g., registered nurses) work individually with clients at an outpatient mental health clinic to coach, connect, and coordinate their primary care services [10]. The core elements of PCARE are care coordination and patient activation. The health-care manager serves as a bridge between clients, primary care and mental health providers to ensure clients’ preventive primary care needs (e.g., screenings) are managed. For clients, the health-care manager serves as an advocate and coach connecting them to primary care services and helping them develop the knowledge and skills to actively participate in their own health care [13]. For primary care and mental health providers, the health-care manager bridges the gap between the mental health-care and primary care sectors by making sure clients’ medical information is shared across systems of care. The health-care manager facilitates clients’ care by monitoring their health and alerting providers when preventive primary care services are needed, following treatment guidelines for preventive primary care [14] and cardiovascular health [15]. The goals of this intervention are to increase the receipt of preventive primary and cardiovascular care, patient activation and health-related quality of life, and reduce risks for cardiovascular disease.

### Intervention adaptation method

We used the collaborative intervention planning framework [3] to inform PCARE adaptation. Following CBPR principles, the framework brings together researchers and community stakeholders through the creation of a community advisory board (CAB) to work on a shared health concern and direct the adaptation process. A CAB provides valuable input about the cultural values, social norms, and community practices and strengths that informs the tailoring of interventions to community realities [16]. The goals of our CAB were the following: 1) maximize the fit of the intervention to its new context by incorporating community knowledge and 2) involve implementers of the intervention (e.g., social workers, administrators) in the adaptation process to enhance their skills, confidence, and capacity to deliver the intervention [3]. CAB members were invited to be part of this board based on their experiences working with Hispanic clients and because they represented the people who could use and deliver this intervention.

CAB meetings were conducted on a monthly basis, lasted approximately an hour, and included a meeting agenda developed by the research team with input from other CAB members. Meetings were led by the principal investigator, and meeting minutes were taken by a research assistant and distributed via e-mail approximately a week in advance of the next meeting for review and comments. A typical meeting included a discussion of the meeting agenda, approval of pervious meeting minutes, project updates, and completion of an IM task (see below) related to intervention adaptations. CAB members not part of the research team received a $15 gift card honorarium for every meeting they attended.

IM procedures organized the adaptation process used by the CAB. IM is a step-by-step group process that uses core activities (e.g., brainstorming, group discussion) and visual tools (e.g., logic models) to develop a road map that informs the development, adaptation, and implementation of interventions [17]. IM has been successfully used for a range of health issues [18-20]. We used a variety of IM activities and tools to guide our intervention adaptation process. Brainstorming exercises and group discussions were used to engage CAB members in the generation of ideas around targeted questions to inform intervention adaptations (e.g., What cultural factors do we need to incorporate into the intervention in order to make it relevant for Hispanic clients?). We conducted group presentations and discussions of existing data and literature reviews, and reviews of intervention materials to understand the health-care needs of Hispanic clients at our site and how the intervention could meet their needs. The CAB also developed and refined an intervention logic model. These are graphic representations that specify the connections between factors that contribute to the health problem and how intervention activities address these factors to achieve desired outcomes [21] (see Additional file 1). We used matrices of change objectives to analyze intervention objectives, methods, and theoretical components of the intervention in order to identify areas for adaptation (see Additional file 1).

The IM steps used in this project are summarized in Table 1. As part of step 2, the research team in partnership with the CAB conducted a mixed-methods needs assessment at our study site between October 2011 and February 2012 to understand Hispanic clients’ physical health problems and experiences with primary care services. Study design and results are published elsewhere and are briefly described here [22]. We used a purposive sample of 40 Hispanic clients from our local clinic that had a chart diagnosis of an SMI and at least one cardiovascular risk factor (e.g., smoking). Data collection included structured interviews that included standardized instruments (e.g., Patient Activation Measure [23], Patient Assessment of Chronic Illness Care [24], Chronic Disease Self-Efficacy Scale [25]), medical chart abstractions, and five focus groups with a subsample of 24 Hispanic clients. Findings that informed intervention adaptations are discussed in the sections below. Two other steps (developing implementation strategies and testing the feasibility and acceptability of the adapted intervention) are currently underway.

**Table 1:** **Summary of the collaborative intervention planning steps for intervention adaptations**

**Step**		**Objectives**	**Number of meetings**	**Activities**	**Products**
1	Setting the stage	• Foster partnership and collaboration	4	Icebreaker activities, mission statement exercises, and group discussions	Mission statement
		• Clarify CAB members’ roles and responsibilities			
		• Introduce project aims, intervention adaptation process, and health-care manager intervention			
2	Problem analysis and needs assessment	• Identify the health-care needs of Hispanic clients	6	Brainstorming exercises, group discussions, development of a logic model, mixed-methods needs assessment that included 40 structured interviews and chart abstraction with Hispanic clients with SMI and at risk for cardiovascular disease and 5 client focus groups	Logic model and needs assessment findings
		• Discuss how the intervention may or may not address these needs			
		• Identify areas for intervention adaptations			
3	Review of intervention objectives and theoretical foundations	• Review the objectives, methods, materials, and theoretical foundations of the intervention	8	Group discussions and review of intervention components, change objective tables, and intervention’s logic model	Revised logic model and change objective tables of adapted intervention
		• Identify specific adaptation to intervention content or delivery			
4	Development of intervention adaptations	• Incorporate adaptations into the intervention manual and materials	8	Review of intervention manual and materials and group discussions	Intervention manual and materials and training curriculum
		• Finalize adapted intervention			

## Results

### CAB members’ participation

The CAB included a primary care physician, the clinic’s director (a psychiatrist), several mental health providers from our site (a social worker, an RN, and a peer specialist), and members of our research team (principal investigator and two research assistants). Intervention adaptation activities for steps 1 to 4 occurred in a 2.9 year period from May 2011 to December 2013. During this time, the CAB met approximately on a monthly basis at the local clinic for a total of 26 meetings. CAB members’ attendance is presented in Table 2; it ranged from 46% to 100% with the highest attendance reported by the members of the research team, social worker, and primary care physician.

**Table 2:** **Community advisory board meeting attendance by year**

**Members**	**Year 1 (** ***N*** **= 6)** ^**a**^		**Year 2 (** ***N*** **= 11 )** ^**a**^		**Year 3 (** ***N*** **= 9)** ^**a**^		**Total (** ***N*** **= 26)**	
	***N***	**%**	***N***	**%**	***N***	**%**	***N***	**%**
Primary care physician	5	83	11	100	8	89	24	92
Social worker	6	100	11	100	8	89	25	96
Nurse	3	50	10	91	8	89	21	81
Peer specialist	5	83	7	64	4	44	16	62
Clinic director^b^	0	0	4	36	8	89	12	46
Principal investigator	6	100	11	100	8	89	25	96
Research assistants	6	100	11	100	9	100	26	100

^a^Number of meetings held in the year.

^b^Clinic director was not invited to attend meetings during year 1.

### Intervention adaptation process

#### Step 1: setting the stage

We dedicated the first four meetings to this step conducting a series of icebreaker activities and dialogs, such as sharing personal reflections about CAB members’ experiences and thoughts about the project. These activities enabled members to get to know one another, discuss their expectations and aspirations, and learn about the project. A key activity in this step was the development of a mission statement. This was an iterative process initiated with a worksheet in which CAB members answered targeted questions (e.g., What does the CAB wants to accomplish?) and then discussed their responses as a group. The research team summarized everyone’s responses and drafted different versions of the statement. The CAB reviewed these drafts and came to a consensus through a group discussion on the final mission statement (see Additional file 1).

#### Step 2: problem analysis and local needs assessment

Six meetings were dedicated to this step. Following IM practices, the CAB’s analysis 1) incorporated multiple perspectives (e.g., research evidence, clinical experience, community knowledge) to understand the health problem; 2) balanced the needs and strengths of the population and setting; 3) used a multilevel ecological approach to identify barriers and facilitators of health; and 4) used logic models as an analytical tool. The following questions informed our analysis: What individual and/or environmental factors place Hispanic clients at high risk for cardiovascular diseases? What existing resources can be used to address these health problems? What cultural factors do we need to incorporate into the intervention to make it relevant for Hispanic clients? What adaptations are needed to help social workers deliver this intervention? Brainstorming activities and group discussions were used to answer these questions. For example, CAB members were given index cards to write down any factors they thought placed Hispanic clients at high risk for CVD. These cards were placed on a dry-erase board and organized along ecological levels to generate a discussion of how PCARE could address these factors. The research team then developed a logic model visually illustrating the CAB’s ideas. The logic model was presented to the CAB to generate further ideas (see Additional file 1).

The CAB also provided input to the design of the needs assessment conducted by the research team by reviewing recruitment materials, study procedures, and data collection instruments. CAB members were involved in the interpretation of needs assessment findings and co-authored a peer-reviewed publication [22].

Results from these activities and the needs assessments indicated that the health problems of Hispanic clients at the local clinic mirror the medical needs of people with SMI with elevated rates of common chronic medical conditions. CAB members also talked about the importance of addressing language barriers when serving Hispanic clients by employing bilingual health-care managers and using culturally and linguistically appropriate patient education materials. Moreover, the needs assessment findings indicated that Hispanic clients at the local clinic tended to report low scores on the Patient Assessment of Chronic Illness Care scale which asses clients’ perspectives of the quality of chronic illness care received from primary care providers in the past 6 months [24] and low scores on the patient activation measure [23] and the chronic disease self-efficacy scale [25]. These findings confirmed that Hispanic patients at our clinic would benefit from health-care manager interventions, like PCARE, since the core elements of this intervention focus on improving care coordination, goal setting, patient activation, and self-management behaviors [22].

In contrast to the original sample used to test PCARE, which tended not to be connected to primary care and was subsequently connected to these services via the intervention, the majority of Hispanic clients at the local clinic had visited a primary care physician during the previous 12 months and were seen in 18 different primary care clinics ranging from large federally qualified health centers to small private doctors’ offices [22]. This suggested that the care coordination efforts in our community were more complex and required adaptations to the care coordination tools specified in PCARE.

We also uncovered through our needs assessment that interpersonal aspects of care, such as the nature of the client-provider relationship, shaped Hispanic clients’ primary care experiences and revealed important insights about Hispanic clients’ preferences and values. Hispanic cultural norms like *personalismo* (being warm and personable; showing that personal ties outweigh formal, institutional connections), *respeto* (respect), and *dignidad* (dignity), were valued by our Hispanic clients, linked to positive experiences with primary care and associated with higher scores in the clients’ assessments of the quality of chronic illness care, patient activation, and self-efficacy [22]. We found that Hispanic clients valued providers who, during their visits, demonstrated these cultural norms by displaying a warm and genuine interest and familiarity about patients’ everyday lives beyond medical issues. These findings indicated that these distinct cultural norms should be integrated into the health-care manager intervention, particularly during the assessment and engagement phase, in order for health-care managers to demonstrate a welcoming, trusting, and respectful culturally compatible relationship with the clients. Overall, step 2 enabled the CAB to corroborate the need for a health-care manager intervention at our local clinic and helped identify key areas for intervention adaptations given unique cultural factors and local circumstances.

#### Step 3: review of intervention to identify program adaptations

Eight CAB meetings were dedicated to this step. During these meetings, the CAB conducted critical reviews of each of the intervention’s core components, activities, and methods in order to identify areas for adaptation. These reviews were informed by the needs assessment findings, existing literature, and CAB members’ clinical and personal experiences. To facilitate this analysis, the research team developed and presented matrices of change objectives that specified the performance objectives (e.g., prepare client for his/her primary care visit), determinants of the objectives (e.g., cultural norms, skills), and the methods and/or activities proposed in the intervention to accomplish the performance objective (e.g., health-care manager rehearses with client how to ask his/her primary care doctor questions during his/her visit) (see Additional file 1). CAB’s discussions of these matrices were intended to help members dissect each element of the intervention in order to understand how the intervention works and achieves its’ desired outcomes and help identify areas to customize the delivery and/or content of the intervention without diluting its core components. The CAB also reviewed the intervention’s logic model to further identify and discuss areas for adaptation. Intervention adaptations and how these were integrated into the intervention are described in the next section.

#### Step 4: development of intervention adaptations

Eight CAB meetings were dedicated to this step. The research team incorporated all the recommendations generated in step 3 into the intervention manual. In this step, the CAB reviewed, discussed, and refined these adaptations. The adaptations that emerged from step 3 and incorporated into the intervention in step 4 are summarized in Table 3 and are organized across two domains: cultural and provider adaptations.

**Table 3:** **Summary of intervention adaptations**

**Intervention domain adapted**	**Rationale for adaptation**	**Description of adaptation**
Cultural adaptations		
Health-care manager personnel	• Language is a critical barrier to care for many Hispanic clients with limited English proficiency.	• Use bilingual health-care managers to deliver the intervention.
Client engagement and client health-care manager interactions	• Health-care manager interpersonal skills and interactions with clients need to reflect core cultural norms valued and preferred by Hispanic clients (e.g., *personalismo, respeto, dignidad*) in order to engender their trust and enhance their engagement.	• Added a section to the intervention manual discussing the importance and rationale for incorporating these cultural norms into health-care manager interpersonal skills and interactions with clients.
		• Added examples in the intervention manual of the type of health-care manager behaviors that demonstrate and reflect each of these cultural norms in their interactions with clients.
Assessment	• Health-care manager assessments need to include the systematic collection of cultural information that can be used to understand Hispanic clients’ perspectives of their health problems, past and present help-seeking, and self-management behaviors, fears, and preferences for care.	• Added the DSM-5 Cultural Formulation Interview adapted for health problems to the assessment protocol used in the initial health-care manager sessions.
Clients’ health education materials	• Client education materials need to be available in English and Spanish and include formats that are relatable, engaging, and relevant to a Hispanic audience.	• Added clients’ health education materials available in Spanish from national organizations (American Diabetes Association, American Heart Association) and health- related *fotonovelas*.
Clients’ activation	• Lack of knowledge and awareness are critical barriers that negatively impact Hispanic clients’ involvement in their own health care and in self-management behaviors of their health conditions.	• Added the personal health record (PHR) as a client education and activation tool and to help facilitate the sharing of medical information between clients and their primary care and mental health providers.
	• Cultural norms associated with deference to authority figures can negatively impact Hispanic clients’ involvement in their medical visits and contribute to clients taking a passive stance toward their primary care physicians.	• Included a client activation checklist to the PHR to help clients’ prepare for their visits with their primary care doctors and be more active during these visits.
	• The multiple stresses and demands that Hispanic clients face in their everyday lives can be overwhelming and create serious barriers for coping and managing their health conditions	• Added a problem solving module to enhance clients’ problem solving skills to cope with their health issues.
Provider adaptations		
Preventive care tracking tool	• Social workers may lack basic medical knowledge about preventive primary and cardiovascular care guidelines for adults, and how to interpret basic lab values for cardiometabolic indicators.	• Modified the preventive care tracking tool by adding basic medical information to facilitate the tracking, interpretation, and coordination of preventive primary care services and cardiovascular care.
Care coordination plan	• Care coordination at our local clinic is more complex than in the original PCARE trial given that our clients receive primary care services from multiple primary care clinics in the community.	• Added a care coordination plan to assist health-care managers tackle the local complexities of coordinating care with multiple doctors and community clinics
		• The plan focuses on developing clear lines of communication to share information about clients’ care and reduce care coordination barriers.
Training curriculum	• Since the original PCARE was delivered by RNs, a training curriculum was needed for our new provider group (social workers)	• A training curriculum consisting of four 3 hour sessions was developed for social workers. It included didactic modules, review and discussion of the program’s manual, role playing activities to practice core health-care managers skills, learning how to read and interpret lab results for cardiometabolic indicators (e.g., lipid panel), and learning how to take simple anthropometric measurements (e.g., weight, blood pressure, waist circumference).

Cultural adaptations focused on addressing language barriers, increasing patient engagement, systematically assessing cultural factors that can influence Hispanic clients’ health-care experiences and enhancing clients’ health knowledge, activation, and problem solving skills. Based on the needs assessment findings, it was deemed essential to use bilingual health-care managers to reduce language barriers. Health-care manager relationship and interactions with the clients also needed to reflect core cultural norms (e.g., *personalismo*, *respeto*) associated with positive primary care experiences and valued by Hispanic clients. A section was added to the intervention manual that defined these cultural norms and provided examples of techniques health-care managers can use to reflect these cultural norms in their interactions with the clients. For example, health-care managers are instructed to show *respeto* (respect) by addressing clients during their first visit by their last name, use terms as *Señor/ra*, and start each session by making polite conversation or *platica* before delving into session activities in order to create a welcoming and warm environment.

Systematically assessing cultural factors during the initial assessment was also deemed critical as it helps increase the validity of the health-care managers’ assessments. The intent of this adaptation was to augment the existing assessment tool with a standardized set of questions that the health-care manager can use to explore clients’ explanatory models of their health conditions, coping strategies, past and present help-seeking attempts, and presence or absence of social supports, fears, and preferences for care. We used an adapted version of the core Cultural Formulation Interview (CFI) developed for the DSM-5 to systematically assess these cultural factors [26].

The core CFI is a semi-structured interview composed of 16 questions that follows a person-centered approach to explore how cultural issues impact a person’s clinical presentation and care [26]. The CFI emphasizes four main assessment domains: 1) cultural definition of the health problem, 2) cultural perceptions of cause, context, and support, 3) cultural factors affecting self-coping and past help-seeking, and 4) cultural factors affecting current help-seeking. Our adapted version of the CFI, the Cultural Formulation Interview for Health (CFI-H), uses the same structure and questions as the original CFI, but focuses on the client’s physical health problems and the social context that surrounds these health issues.

Low health literacy was also seen as a critical barrier for helping Hispanic clients manage their health conditions. CAB’s discussions led to the recommendation that clients’ health education materials be available in English and Spanish, included simple language (e.g., 4th to 6th grade reading level) and visuals (e.g., photographs, colorful graphics) to facilitate Hispanic clients’ comprehension and use of these materials. The CAB recommended that educational materials include formats that are relatable, engaging, and relevant to Hispanic audiences. To this end, we added education materials from national organizations (e.g., American Heart Association) available in English and Spanish and health-related *fotonovelas* to our health education tools. *Fotonovelas* are popular health education tools that use posed photographs or drawings, captions, and soap opera narratives to engage audiences and raise knowledge and awareness about health issues [27].

Cultural adaptations to client activation activities focus on increasing the clients’ knowledge and access to their health information and help counteract cultural norms associated with deference to authority figures, particularly when visiting primary care doctors. This was seen as a critical step since some Hispanic clients with SMI tend to ascribe to social norms dictating that people should respect and not overtly question authority figures (e.g., doctors) in order to preserve social equanimity during these interactions and avoid being perceived as difficult clients [28]. Then, after the medical encounter, the clients exert their autonomy by deciding whether or not to follow their doctors’ treatment recommendations without disclosing their decisions with their clinicians. This external deference to authority can undermine the clients’ involvement in their own medical care by not asking questions or expressing their concerns about their medical conditions or treatments. This passivity during the medical encounter can be misinterpreted by clinicians as demonstrating that the client understands all instructions and is in full agreement with all treatment decisions which can then frustrate clinicians when the client does not adhere to treatment recommendations at later visits.

To address these barriers, the personal health record (PHR) was added to the intervention. PHR is a patient-centered communication and patient activation tool that helps the clients manage transitions between health-care settings and facilitate continuity of care [29]. As a communication tool, the PHR is used to share critical health information relevant to the client’s chronic conditions and CVD risk factors with different providers. As an activation tool, it enhances the clients’ self-management skills by having them review and track health information and prepare for visits and discussions with providers. The PHR is a living document that the health-care manager and client regularly update. The components of the PHR used in our intervention included the following: contact information of the health-care manager, primary care physician, and mental health provider; a list of the client’s health and mental health conditions; an up-to-date list of the client’s medications; information on client’s CVD risk factors (e.g., cholesterol levels); an activation checklist composed of simple tasks and instructions to help the client prepare for a medical visit (e.g., bring a list of your medications to your medical visits); a list of questions the client could ask his/her primary care doctor (e.g., what are the side effects of my medications?); and a section where the client can write questions to ask the primary care doctor.

Lastly, the CAB recommended enhancing the clients’ problem solving skills given their observations that Hispanic clients often felt overwhelmed coping with their health issues because of the multiple stresses and barriers (e.g., financial strain, family problems). Problem solving is a core self-management skill for people living with chronic medical conditions and a central element of self-management programs [30]. To this end, we added a problem solving module that included a worksheet the health-care managers could use with their clients to apply problem-solving steps and techniques to a particular health-related problem.

Provider adaptations focused on facilitating social workers’ delivery of the intervention by modifying the preventive primary care tracking tool, developing a local care coordination plan, and developing a training curriculum for social workers. Social workers may lack basic medical knowledge about preventive primary care and cardiovascular care guidelines for adults and may not know how to interpret lab values for common cardio-metabolic indicators (e.g., lipid panels). To address these issues, the CAB modified the original PCARE preventive primary care tool by adding simple explanations for each guideline, including normal ranges for lab values, when indicators should be tracked (e.g., annually) and instructions the health-care manager can follow when a guideline is not met or a lab value is above the normal threshold follow (e.g., alert client’s primary care provider).

Since the care coordination at our local clinic was found to be more complex than in the original PCARE trial, we added a local tool to the intervention that health-care managers could use to plan, organize, and facilitate their care coordination activities. This care coordination planning tool focused on enabling the health-care manager to clearly define and establish lines of communication with the clients’ primary care and mental health providers based on the providers’ preferences for sharing information.

Lastly, a training curriculum for social workers was developed to expand the use of this health-care manager intervention to this provider group. The training curriculum focused on helping social workers increase their knowledge and skills in taking on this new role and enable them to practice and receive feedback from trainers to build their confidence in delivering this intervention. The training curriculum delivered by the research team consists of four 3-h sessions that include didactic modules, review and discussion of the intervention’s manual, role playing activities with constructive performance feedback, and discussion groups.

## Discussion

In this study, we described the application of the collaborative intervention planning framework to engage researchers and stakeholders in the adaptation of an existing health-care manager intervention for Hispanic clients with SMI and social workers. Consistent with other intervention adaptation models [31-33], our collaborative framework involved multiple stakeholders in the adaptation process, used formative research methods to understand the needs of the new population and context of practice and identify adaptation targets, followed a series of adaptation steps, and documented the rationale for each intervention adaptation. Two critical strengths of our approach were the use of CBPR principles and IM procedures.

The following CBPR principles informed our approach: 1) co-learning between researchers and stakeholders, 2) valuing multiple sources of knowledge to inform intervention adaptations, 3) involving stakeholders and researchers in all phases of the process, 4) employing a strength-based ecological perspective to all project activities, and 5) building the stakeholders’ capacity to use the adapted intervention [34]. IM procedures provided the logistical roadmap to systematically analyze each intervention component and its fit with the new client population, provider group, and local setting. It helped addressed the blackbox problem common in many adaptation models in which the procedures used to carry out the adaptation process with stakeholders are often vague and unspecified, thus difficult to replicate by other groups.

An important step in transporting an existing intervention into a new setting and population is to determine whether the new intervention meets the health-care needs of the local population. This important decision was addressed in step 2 through our mixed-methods needs assessment and the CAB’s analysis of these findings which corroborated that clients at our local clinic would benefit from an intervention like PCARE as the core elements of this intervention focus on improving care coordination, goal setting, patient activation, and self-management behaviors. The application of our framework, particularly steps 2 to 3, was instrumental for determining the type of intervention adaptations needed to prepare PCARE to our local context. These steps indicated that the adaptations should not alter or delete the core elements of PCARE, but instead focused on cultural and provider-level issues that would enhance the fit and relevance of the intervention to the cultural characteristics of Hispanic clients and help facilitate social workers’ delivery of the intervention.

We identified a combination of surface and deep-level cultural adaptions. Surface-level adaptations involve customizing intervention content, messages, and approaches to the observable cultural characteristics of the local patient population in order to enhance the intervention’s appeal, receptivity, and feasibility [35]. Our surface adaptations (e.g., using bilingual health-care managers, adding health education materials in Spanish and English) were central for making the intervention and its components more acceptable to Hispanic clients. Deep-level adaptations entail incorporating clients’ cultural values, norms, and preferences into the intervention in order to enhance the intervention’s salience and impact [35]. Our deep-level adaptations addressed three areas. First, we incorporated specific cultural values and norms (e.g., *personalismo*) into health-care manager-patient interactions in order to make these interactions more culturally responsive. Past studies have found that these cultural norms are critical for building trust between Hispanic clients and providers and enhance treatment engagement [36]. Second, we added a patient activation checklist to the PHR to counteract common cultural norms in the Hispanic community dictating that people should show deference to authority figures in order to avoid disagreements during the medical encounter, which can negatively impact clients’ health-care experiences [28]. Lastly, we added the CFI-H to help health-care managers systematically assess clients’ sociocultural context. As a patient-centered assessment, the CFI-H conveys to clients that the health-care manager is interested in understanding their illness narrative and reflects the cultural value of *personalismo* as it grounds the assessment in the individual’s experiences and honors Hispanic clients’ expectations of having a personalized, informed, caring, and warm relationship with their providers [36].

The application of our collaborative intervention planning framework enabled us to identity provider-level adaptations to facilitate social workers’ delivery of this intervention. Social workers deliver most of the mental health care in the U.S. [37], and can effectively serve as care managers for mental and physical illnesses [38-41]. Provider-level adaptations were intended to add new knowledge and skills necessary to help social workers monitor and manage the common preventive primary care and cardiovascular issues that impact people with SMI. Expanding the use of an intervention like PCARE to social workers in outpatient mental health clinics is critical for enhancing the reach of this intervention, particularly as health-care managers are central to the medical homes being implemented through health-care reform in the US [2].

## Conclusions

Health interventions, like PCARE, could be widely used to improve the health of people with SMI. To increase the reach of these interventions in the public mental health system and among racial and ethnic communities, local adaptations may be needed. We applied the collaborative intervention planning framework to customize the use of PCARE to our local community. In this framework, CBPR principles served to foster a partnership between researchers and community stakeholders, and IM provided the means for putting this partnership into action. Future studies are needed to replicate the use of this framework with other populations, settings, and interventions and test the feasibility, acceptability, and outcomes of the adapted intervention. Reducing health disparities in people with SMI requires community engagement, particularly when preparing interventions to be used with new client and provider groups. Our study illustrates one approach that can be used to involve stakeholders in the intervention adaptation process from the very beginning to enhance the transportability of health-care manager interventions to address the health and health-care disparities faced by people with SMI.

## Additional file

Additional file 1:
**Mission statement, logic model, and matrix.**

## Abbreviations

CAB: Community advisory board
CBPR: Community-based participatory research
CFI: Cultural formulation interview
CFI-H: Cultural formulation interview for health
IM: Intervention mapping
CVD: Cardiovascular disease
PCARE: Primary Care, Access, Referral and Evaluation program
PHR: Personal health record
RN: Registered nurse
SMI: Serious mental illness

## References

[1] Institute of Medicine (2006). Improving quality of health care for mental and substance use conditions: quality chasm series. Book Improving Quality of Health Care for Mental and Substance use Conditions: Quality Chasm Series.

[2] Alakeson V, Frank RG, Katz RE (2010). Specialty care medical homes for people with severe, persistent mental disorders. Health Aff (Millwood).

[3] Cabassa LJ, Druss B, Wang Y, Lewis-Fernandez R (2011). Collaborative planning approach to inform the implementation of a healthcare manager intervention for Hispanics with serious mental illness: a study protocol. Implement Sci.

[4] Colton CW, Manderscheid RW (2006). Congruencies in increased mortality rates, years of potential life lost, and causes of death among public mental health clients in eight states. Prev Chronic Dis.

[5] Druss BG, Zhao L, Von Esenwein S, Morrato EH, Marcus SC (2011). Understanding excess mortality in persons with mental illness: 17-year follow up of a nationally representative US survey. Med Care.

[6] Hellerstein DJ, Almeida G, Devlin MJ, Mendelsohn N, Helfand S, Dragatsi D, Miranda R, Kelso JR, Capitelli L (2007). Assessing obesity and other related health problems of mentally ill Hispanic patients in an urban outpatient setting. Psychiatr Q.

[7] Kato MM, Currier MB, Gomez CM, Hall L, Gonzalez-Blanco M (2004). Prevalence of metabolic syndrome in Hispanic and Non-Hispanic patients with schizophrenia. Prim Care Companion J Clin Psychiatry.

[8] Henderson DC, Nguyen DD, Copeland PM, Hayden DL, Borba CP, Louie PM, Freudenreich O, Evins AE, Cather C, Goff DC (2005). Clozapine, diabetes mellitus, hyperlipidemia, and cardiovascular risks and mortality: results of a 10-year naturalistic study. J Clin Psychiatry.

[9] Druss BG (2007). Improving medical care for persons with serious mental illness: challenges and solutions. J Clin Psychiatry.

[10] Druss BG, von Esenwein SA, Compton MT, Rask KJ, Zhao L, Parker RM (2010). A randomized trial of medical care management for community mental health settings: the Primary Care Access, Referral, and Evaluation (PCARE) study. Am J Psychiatry.

[11] Rogers EM (1995). Diffusions of Innovations.

[12] New York City Department of Health and Mental Hygiene (2006). Community health profile: Central Harlem and Inwood Washington Heights. Book Community Health Profile: Central Harlem and Inwood Washington Heights.

[13] Hibbard JH, Tusler M (2007). Assessing activation stage and employing a “next steps” approach to supporting patient self-management. J Ambul Care Manage.

[14] United States Preventive Services Task Force (2010). The Guide to Clinical Preventive Services 2010–2011: Recommendations of the US Preventive Services Task Force.

[15] American Diabetes Association, American Psychiatric Association, American Association of Clinical Endocrinologists, North American Association for the Study of Obesity (2004). Consensus development conference on antipsychotic drugs and obesity and diabetes. J Clin Psychiatry.

[16] Devieux JG, Malow RM, Rosenberg R, Jean-Gilles M, Samuels D, Ergon-Perez E, Jacobs R (2005). Cultural adaptation in translational research: field experiences. J Urban Health.

[17] Tortolero SR, Markham CM, Parcel GS, Peters RJ, Escobar-Chaves SL, Basen-Engquist K, Lewis HL (2005). Using intervention mapping to adapt an effective HIV, sexually transmitted disease, and pregnancy prevention program for high-risk minority youth. Health Promot Pract.

[18] Fernandez ME, Gonzales A, Tortolero-Luna G, Partida S, Bartholomew LK (2005). Using intervention mapping to develop a breast and cervical cancer screening program for Hispanic farmworkers: Cultivando La Salud. Health Promot Pract.

[19] Belansky ES, Cutforth N, Chavez RA, Waters E, Bartlett-Horch K (2010). An adapted version of intervention mapping (AIM) is a tool for conducting community-based participatory research. Health Promot Pract.

[20] Schmid AA, Andersen J, Kent T, Williams LS, Damush TM (2010). Using intervention mapping to develop and adapt a secondary stroke prevention program in veterans health administration medical centers. Implement Sci.

[21] Bartholomew LK, Parcel GS, Kok G, Gottlieb NH (2006). Planning Health Promotion Programs: Intervention Mapping.

[22] Cabassa LJ, Gomes AP, Meyreles Q, Capitelli L, Younge R, Dragatsi D, Alvarez J, Nicasio A, Druss B, Lewis-Fernandez R (2013). Primary health care experiences of Hispanics with serious mental illness: a mixed-methods study. Adm Policy Ment Health.

[23] Hibbard JH, Mahoney ER, Stockard J, Tusler M (2005). Development and testing of a short form of the patient activation measure. Health Serv Res.

[24] Glasgow RE, Whitesides H, Nelson CC, King DK (2005). Use of the Patient Assessment of Chronic Illness Care (PACIC) with diabetic patients: relationship to patient characteristics, receipt of care, and self-management. Diabetes Care.

[25] Lorig K, Stewart A, Ritter P, González V, Laurent D, Lynch J (1996). Outcome Measures for Health Education and other Health Care Interventions.

[26] Lewis-Fernandez R, Aggarwal NK, Baarnhielm S, Rohlof H, Kirmayer LJ, Weiss MG, Jadhav S, Hinton L, Alarcon RD, Bhugra D, Groen S, van Dijk R, Qureshi A, Collazos F, Rousseau C, Caballero L, Ramos M, Lu R (2014). Culture and psychiatric evaluation: operationalizing cultural formulation for DSM-5. Psychiatry.

[27] Cabassa LJ, Molina GB, Baron M (2012). Depression fotonovela: development of a depression literacy tool for Latinos with limited English proficiency. Health Promot Pract.

[28] Cabassa LJ, Siantz E, Nicasio A, Guarnaccia P, Lewis-Fernandez R (2014). Contextual factors in the health of people with serious mental illness. Qual Health Res.

[29] Coleman EA, Smith JD, Frank JC, Min SJ, Parry C, Kramer AM (2004). Preparing patients and caregivers to participate in care delivered across settings: the care transitions intervention. J Am Geriatr Soc.

[30] Lorig KR, Holman HR (2003). Self-management education: history, definition, outcomes, and mechanisms. Ann Behav Med.

[31] Domenech Rodriguez MM, Baumann AA, Schwartz AL (2011). Cultural adaptation of an evidence based intervention: from theory to practice in a Latino/a community context. Am J Community Psychol.

[32] Ferrer-Wreder L, Sundell K, Mansoory S (2012). Tinkering with perfection: theory development in the intervention cultural adaptation field. Child Youth Care Forum.

[33] Castro FG, Barrera M, Martinez CR (2004). The cultural adaptation of prevention interventions: resolving tensions between fidelity and fit. Prev Sci.

[34] Israel BA, Schulz AJ, Parker EA, Becker AB (1998). Review of community-based research: assessing partnership approaches to improve public health. Annu Rev Public Health.

[35] Resnicow K, Baranowski T, Ahluwalia JS, Braithwaite RL (1999). Cultural sensitivity in public health: defined and demystified. Ethn Dis.

[36] Organista KC (2007). Solving Latino Psychosocial and Health Problems: Theory, Practice, and Population.

[37] Proctor EK (2004). Research to inform mental health practice: social work’s contribution. Soc Work Res.

[38] Ell K, Katon W, Cabassa LJ, Xie B, Lee PJ, Kapetanovic S, Guterman J (2009). Depression and diabetes among low-income Hispanics: design elements of a socioculturally adapted collaborative care model randomized controlled trial. Int J Psychiatry Med.

[39] Ell K, Quon B, Quinn DI, Dwight-Johnson M, Wells A, Lee PJ, Xie B (2007). Improving treatment of depression among low-income patients with cancer: the design of the ADAPt-C study. Gen Hosp Psychiatry.

[40] Ell K, Unutzer J, Aranda M, Gibbs NE, Lee PJ, Xie B (2007). Managing depression in home health care: a randomized clinical trial. Home Health Care Serv Q.

[41] Ell K, Vourlekis B, Lee PJ, Xie B (2007). Patient navigation and case management following an abnormal mammogram: a randomized clinical trial. Prev Med.

