# Supplementary material for: Using the collaborative intervention planning framework to adapt a health-care manager intervention to a new population and provider group to improve the health of people with serious mental illness
Source: Implement Sci. 2014 Nov 30;9:178. doi: 10.1186/s13012-014-0178-9 (PMC4255430; doi:10.1186/s13012-014-0178-9)
Supplement: Additional file 1: — Mission statement, logic model, and matrix. [file 13012_2014_178_MOESM1_ESM.doc]

**Additional File 1**

**Mission Statement**

The mission of the Community Advisory Board for Health Integration is to develop and implement culturally appropriate evidence-base health care interventions to improve the physical health and quality of life of patients at the [Local Clinic Name]. To achieve these goals, the board will focus on four main areas:

1. Increase patients’ access to primary care
2. Improve the coordination of medical and mental health care for people with serious mental illness, including systematically monitoring the physical health status of patients
3. Improve the knowledge and skills of mental health clinicians and primary care providers so that they are prepared to address the physical health needs of patients with serious mental illness
4. Empower patients and their families to become active participants in their own medical care and health promotion activities.

**Adapted Health Care Manger Intervention Logic Model**

Intervention Components

Outcomes

Problem Factors

System-Level

Fragmentation of:

1. Services

2. Personnel

3. Finance

4. Organizations

Care Coordination

1. **Provider outreach**

2. **Care coordination and patient navigation**

3. **Care monitoring and management**

- Personal health record

Proximal Outcomes

1. Patient activation (↑)

2. Health self-efficacy (↑)

3. Receipt of preventive primary care (↑)

4. Receipt of quality cardio-metabolic care (↑)

5. General health-status (↑)

6. Patient Assessment of Chronic Illness Care (↑)

Distal Outcomes

1. Health and Mental Health-Related Quality of Life (↑)
2. Cardio- vascular Risk (↓)

Environmental-Level

1. Under-resourced communities

2. Poor access and availability of care

3. Variable quality of primary care

Assessment and Planning

1. **Comprehensive health assessment**

- Cultural formulation interview for health

2. **Treatment Plan for preventive primary care and CVD risk reduction**

Provider-Level

1. Training

2. Professional boundaries

3. Stigma

4. Cultural competence

Client-Level

1. Health beliefs & knowledge

2. Cultural norms

3. Health literacy

4. Competing demands

Client Activation

1. **Health education**

2**. Action planning**

3. Problem -solving

4. Primary Care Visit checklist

Note: PCARE core elements are **bolded**. Intervention adaptations are underlined.

**Example of matrix illustrating the link between change objectives, intervention methods, strategies and intervention components**

| **Change Objectives** | **Methods** | **Strategies** | **Intervention Components** |
| --- | --- | --- | --- |
| **Care Manger Assessment and Planning (AP)** | | | |
| AP1) Complete comprehensive health assessment focus on preventive primary care and cardiovascular health | - Information - Cues to action | - Health care manager (HCM) conducts a comprehensive and standardized physical health assessment on every client on their first visit - HCM conducts the cultural formulation interview for Health on every client on their first visit - HCM charts assessment and develops personal health record - HCM discusses assessment with client, mental health provider and primary care provider | - Initial physical health assessment - Cultural formulation interview for health - Personal health record |
| AP2) Develop treatment plan for preventive primary care and cardiovascular risk reduction | - Goal setting - Monitoring - Cues to action | - HCM works with client to identify initial clients’ health goals and develops first action plan related to preventive primary care and cardiovascular risk reduction - HCM fills out preventive care tracking tool and alerts client, mental health provider, and primary care providers of necessary monitoring and management services for preventive primary and cardiovascular care | - Action plan - Preventive care tracking tool |
| **Care Coordination (CC)** | | | |
| CC1) Improve communication between providers | - Information - Cues to action - Monitoring | - HCM initiates contact with client’s primary care providers and introduces project and HCM role - HCM develops tailored communication plan with client’s primary care and mental health care providers - CM tracks communication with clients’ providers | - Letters and scripts - Personal health record - Care coordination plan |
| CC2) Assist patient navigation | - Information - Modeling - Guided practice - Goal setting | - HCM initial assessment - HCM assist client prepare and schedule visit with primary care doctors - HCM accompanies patients to visits, if necessary | - Personal health record - Action planning - Problem solving module |
| CC3) Facilitate monitoring, management, and coordination of patient health care needs | - Information - Cues to action - Monitoring | - HCM uses preventive care tracking care tool to monitor and manage client’s preventive primary care and cardiovascular care needs - HCM updates personal health record based on new health information and shares the new health information with clients, mental health providers, and primary care providers | - Personal health record - Preventive care tracking tool |
| **Patient Activation (PA)** | | | |
| PA1) Patient education and awareness | - Information - Cues to action | - HCM uses patient education materials and individual sessions to raise client’s awareness and self-efficacy to address personal goals for preventive primary care and reduce cardiovascular risk. | - Patient educational materials - Health-related *Fotonovelas* |
| PA2) Action planning | - Cues to action - Guided practice - Modeling | - HCM uses action planning steps to help clients work on their self-identified preventive primary care and cardiovascular health goals - HCM uses problem solving steps to help clients work towards their identified preventive primary care and cardiovascular health goals. | - Action planning worksheet - Problem solving worksheet |
| PA3) Primary care visit activation | - Information - Guided practice - Modeling | - HCM prepares and coaches clients to be proactive and engaged during their primary care visit - HCM models and practices with the client how to use the personal health record during primary care visit,. - HCM uses problem solving worksheet to discuss how to address barriers for engaging in primary care visits. | - Personal health record - Problem solving worksheet |
